# SARS-CoV-2 vaccine development and how Brazil is contributing

**DOI:** 10.1590/1678-4685-GMB-2020-0320

**Published:** 2021-04-02

**Authors:** Alex I. Kanno, Mayra M.F. Barbosa, Luana Moraes, Luciana C.C. Leite

**Affiliations:** 1 Instituto Butantan, Laboratório de Desenvolvimento de Vacinas, São Paulo, SP, Brazil.; 2 Universidade de São Paulo, Programa de Pós-Graduação Interunidades em Biotecnologia, São Paulo, Brazil.

**Keywords:** SARS-CoV-2, vaccine, immunization strategies

## Abstract

The SARS-CoV-2 coronavirus pandemic calls for coordinated efforts by the scientific community for the development of vaccines. The most advanced strategies have focused on modifications of technologies that were already under development for other viruses, such as SARS, MERS, and even Influenza. Classic and new technologies, such as inactivated and attenuated viruses (non-replicative and replicative), DNA and mRNA vaccines, and nanoparticles containing SARS-CoV-2 antigens, are some of the strategies currently investigated. Although there is a very high expectation for the effectiveness of the most advanced vaccine candidates, there are still no established correlates of protection. Previous experience in vaccine development for other pathogens shows that differences in vaccine formulation can result in diverse immune responses and consequently, different protective properties. Therefore the importance of continuing investigations on a broad range of strategies. Expertise in vaccine development in Brazil was refocused to the new coronavirus. Impressive collaboration between institutions will support further developments until we have available a safe, effective, and economically viable vaccine. Established competence and collaborations will allow preparedness for future challenges and can also be used to address local issues as neglected infectious diseases.

## Importance of vacines in disease control

Vaccination is one of the most important human achievements in biomedical sciences. It has successfully reduced the burden of infectious diseases worldwide. According to the World Health Organization (WHO), the benefits of vaccination go beyond the individual protection provided by the vaccine against the targeted pathogen. Ideally, it targets the complete eradication of the pathogen so it cannot re-emerge. The eradication of smallpox allowed the discontinuation of routine immunization. Furthermore, vaccination can be used to control mortality, morbidity, and mitigate disease severity (Greenwood, 2014). Other advantages include the protection of the non-vaccinated population (herd immunity), against related and unrelated diseases, healthcare savings, prevention of antibiotic resistance, and extension of life expectancy (Andre et al., 2008).

The first cases of an “unknown cause” of pneumonia were reported in December 2019, to the WHO office in China. By January, it had been identified as a new coronavirus (SARS-CoV-2, leading to COVID-19 disease) and crossed the Chinese borders. It was declared a pandemic in March. The rate at which the SARS-CoV-2 virus spread through the world and shutdown country borders, industries and local businesses, only reinforces the importance of vaccines in disease control. The cost-effectiveness of a vaccine in this scenario is indisputable. The worldwide efforts to develop an effective vaccine against SARS-CoV-2 and protect against the COVID-19 disease, have driven many collaborations, along with unprecedented governmental support, leading to hundreds of strategies in pre-clinical and clinical evaluations and so far, eight vaccine candidates are in phase III clinical trials, the last before registration (WHO, 2020a).

## The race for vaccine development

The outbreak of COVID-19 has led to a global race for the development of vaccines and treatments in record time. The initiatives involve hundreds of countries, public-private partnerships, multinational pharmaceuticals, and biotech companies. The Landscape of COVID-19 candidate vaccines as of 12 November 2020 reports 48 candidates in clinical trials (Table 1) and 164 others in the preclinical stage. Different websites, (WHO, 2020a), (Milken Institute, 2020) and others, provide updated information on the vaccines in development as they progress into clinical trials.

**Table 1 -:** Vaccine candidates currently in Clinical trials.

Vaccine platform	Name	Institution	Country	Route	Details of platform	General safety and Advantages	Clinical Stage	Trial number
Inactivated virus	CoronaVac	Sinovac Biotech	China	i.m.	The SARS-CoV-2 virus inactivation + adjuvant.	Inactivated vaccines used throughout the world with a generally excellent safety profile. Straightforward process; favorable safety and tolerability profile	Ph III	NCT04456595 NCT04582344
	BBIBP-CorV	Sinopharm/Beijing Institute	China	i.m.			Ph III	ChiCTR2000034780
	Unnamed	Sinopharm/Wuhan Institute	China	i.m.			Ph III	ChiCTR2000034780
	BBV152	Bharat Biotech	India	i.m.			Ph III	CTRI/2020/11/028976
	Unnamed (Yunnan)	Chinese Acad. Of Medical Sciences	China	i.m.			Ph I/II	NCT04470609
	QazCovid-in	Research Institute for Biological Safety Problems	Kazakhstan	i.m.			Ph I/II	NCT04530357
	Unnamed	Beijing Minhai Biotech Co Ltd	China	i.m.			Ph I/II	ChiCTR2000039462
Non-replicating viral vector (Adenovirus and MVA)	AZD1222	Oxford/Astra Zeneca	UK	i.m.	Different Adenovirus expr. S glycoprotein ChAdOx1 (Chimp - Oxford), Ad5 (CanSino), Ad26 (J&J), RD-Ad5 (Altimune)	In general, safe and well tolerated; concerns for immunocompromised individuals. Vector used in gene therapy & vaccination. Ad5 and Ad26 - high titer stable stocks. Ad26 - low preexisting antibodies to the vector.	Ph III	ISRCTN89951424
	Ad5-nCov	CanSino Biological	China	i.m./ mucosal			Ph III Ph I	NCT04526990 NCT04552366
	Gam-COVID-Vac	Gamaleya Research Institute	Russia	i.m.			Ph III	NCT04530396
	Ad26.COV2-S	Janssen Pharmaceutical	USA	i.m.			Ph III	NCT04505722
	GRAd-COV2	Pasteur/Thera/LEUKOCARE	Italy	i.m. 1-dose			Ph I	NCT04528641
	hAd5-S-Fusion+N-ETSD	ImmunityBio & NantKwest	USA	s.c.			Ph I	NCT04591717
	VXA-CoV2-1	Vaxart	USA	oral			Ph I	NCT04563702
	MVA-SARS-2-S	Ludwig-Maximilians/Univ. of Munich	Germany	i.m.	Attenuated poxvirus expressing Spike	Safety attested by its use as against smallpox. High immunogenicity including in the lungs.	Ph I	NCT04569383
Replicating viral vector	DelNS1-2019-nCoV-RBD-OPT1	Beijing Wantai Biological Pharmacy/Xiamen Univ.	China	i.n.	Flu-based vaccine expressing RBD	In general, safe and well tolerated	Ph II	ChiCTR2000039715
	rVSV-SARS-CoV-2-S	Israel Institute for Biological Research	Israel	i.m.	Vesicular Stomatitis Virus (VSV) expressing Spike	Severely attenuated. No prior immunity. High protein expression	Ph I/II	NCT04608305
	V590	Merck Sharp & Dohme/IAVI	USA	i.m.			Ph I	NCT04569786
	TMV-083	Institut Pasteur/ Themis Bioscience	France	i.m.	Live-attenuated measles vaccine expr. Spike	In general, safe and well tolerated. Vector tested in chikungunya vaccine	Ph I	NCT04497298
DNA	INO-4800	Inovio Pharmaceuticals	USA	i.d. electro	Plasmid/Spike electroporation	Favorable safety and tolerability profile. No DNA vaccines currently in use in humans. Fast design/manufacturing; no cold chain for storage/distribution.	Ph I/II	NCT04336410 NCT04447781
	AG0301-COVID19	Osaka Univ./ AnGes/Takara Bio	Japan	i.m.	Plasmid/ Spike		Ph I/II	NCT04463472 NCT04527081
	ZyCoV-D	Cadila Healthcare Limited	India	i.d.	Plasmid/ M protein		Ph I/II	CTRI/2020/07/026352
	GX-19	Genexine Consortium	South Korea	i.m.	Plasmid/Spike		Ph I/II	NCT04445389
	bacTRL-Spike	Symvivo	Canada	oral	Plasmid/ Trim. Spike in Bifidobacterium		Ph I	NCT04334980
Protein subunit	NVX-CoV2373	Novavax	USA/ Australia	i.m.d	Spike in nanoparticle and Matrix M adjuvant	Platforms showed safety in several clinical trials for influenza and RSV. Well-established combination w/ adjuvants Fast design/production processes	Ph III	NCT04611802
	RBD-Sc dimer	Anhui Zhifei Longcom Biopharmaceutical	China	i.m.d	RBDs in fusion		Ph II	NCT04466085
	KBP-201	Kentucky Bioprocessing	-	i.m.	RBD-based		Ph I/I	NCT04473690
	Unnamed	Sanofi Pasteur / GSK	USA	i.m.	Spike protein		Ph I/I	NCT04537208
	BECOV	Biological E Ltd	India	i.m.	RBD + adjuvant		Ph I/I	CTRI/2020/11/029032
	SCB-2019	Clover Biopharm/GSK/ Dynavax	China	i.m.d	Trim. rSpike		Ph I	NCT04405908
	Covax-19	GeneCure Biotechnologies/ Vaxine/ Medytox	USA/ Australia	i.m.	rSpike with Advax™ adjuvant (polysaccharide)		Ph I	NCT04428073 NCT04453852
	Unnamed	Univ. Queensland/ CSL/ Seqirus	Australia	i.m.	Molecular clamp of viral antigens		Ph I	ACTRN12620000674932 NCT04495933
	MVC-COV1901	Medigen Vaccine Biologics Corporation/NIAID/Dynavax	Taiwan	i.m.	S-2P protein + CpG 1018		Ph I	NCT04487210
	FINLAY-FR-2	Instituto Finlay de Vacunas, Cuba	Cuba	i.m.	RBD conjugated to tetanus toxoid		Ph I	IFV/COR/06
	FINLAY-FR-1	Instituto Finlay de Vacunas, Cuba	Cuba	i.m.	RBD + adjuvant		Ph I	IFV/COR/04 IFV/COR/05
	EpiVacCorona	FBRI SRC VB VECTOR, Rospotrebnadzor, Koltsovo	Russia	i.m.	Peptide		Ph I	NCT04527575
	Unnamed	West China Hospital	China	i.m.	RBD + Alum		Ph I	ChiCTR2000037518
	CoVac-1	University Hospital Tubingen	Germany	s.c.	SARS-CoV-2 HLA-DR peptides		Ph I	NCT04546841
	UB-612	COVAXX / United Biomedical Inc. Asia	Taiwan	i.m.	Spike-RBD multiepitope		Ph I	NCT04545749
VLP	RBD SARS-CoV-2 HBsAg VLP	SpyBiotech/Serum Institute of India	UK/India/ Australia	i.m.	HBsAg VLPs containing RBD	Technology shown safe in Ph III trials for influenza vaccine. Stable, safe, preserves structure of viral particle	Ph I/II	ACTRN12620000817943
	CoVLP	Medicago/GSK	Canada/USA	i.m.	VLP expr Spike w/ adjuvant.		Ph I	NCT04450004
Others	AV-COVID-19	Aivita Biomedical	USA	s.c.	Dendritic cells (DC) loaded w/ SARS-CoV-2 antigens	Platform tested in several trials for cancer. DCs activate innate and adaptive immunity.	Ph I/II	NCT04386252
	Covid-19/aAPC	Shenzhen Geno-Immune Medical Institute	China	s.c.	Artificial APC altered by lentivirus	Cells inactivated for proliferation. Safety tested	Ph I	NCT04299724
	AlloStim	Immunovative Therapies/ Mirror Biologics, Inc	USA	i.d.	Genetically attenuated SARS-CoV-2	Bioengineered live vaccines - generally an excellent safety and tolerability	Ph I/II	NCT04441047
Heterologous protection	BCG Vaccine	Royal Children's Hosp/ Baylor College of Med./ Harvard Univ./ Max Planck Inst./ Hosp. Univ. Dr. Jose E. Gonzalez	UK/ USA/ Germany/ Brazil	i.d.	Live vaccines may confer non-specific effects, reducing morbidity and mortality from other infections	Approved use for humans Known manufacture	Ph IV	NCT04327206 NCT04369794 NCT04439045 NCT04328441 NCT04384549 NCT04348370 NCT04461379
	Polio Vaccine	Bandim Health Project	Republic of Guinea-Bissau	oral			Ph IV	NCT04445428
	MMR Vaccine	Kasr El Aini Hospital, Louisiana State University	USA/ Netherlands/ Egypt	i.m.			Ph IV	NCT04357028 NCT04475081 EudraCT2020-002456-21

Admin., administration; w/, with; exp., expressing; Ph, phase; i.m., intramuscular; i.n.; intranasal; i.d., intradermal; i.m.d, deltoid; electro., electroporation; LNP, lipid nanoparticle; RBD, receptor-binding domain; Trim., Trimeric; rSpike, recombinant Spike protein; Univ., University; Inst., Institute.

**Figure 1 -:**
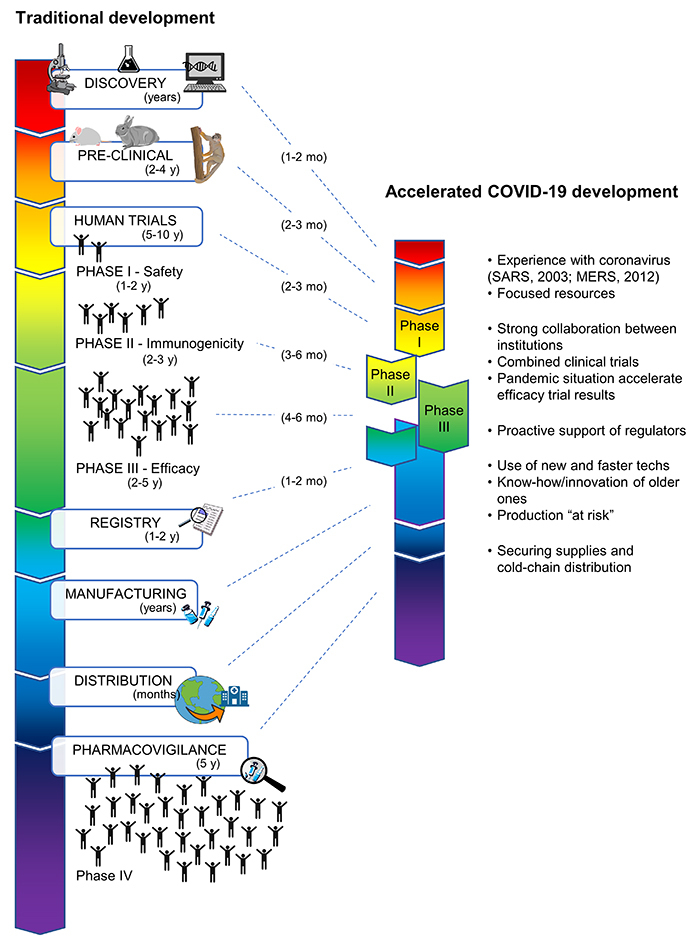
Traditional and accelerated COVID-19 vaccine development. Illustration of the steps and timeline of traditional vaccine development in comparison to the accelerated COVID-19 scheme. Factors that explain the fast-paced progress for COVID-19 are presented.

In general, vaccine development undergoes several steps: discovery, pre-clinical tests, and clinical trials, subdivided into phases I, II, and III, registration and phase IV (Figure 1). The discovery phase comprises the choice of the platform, design of targets, preparation of small batches, and *in vitro* testing. The pre-clinical stage involves target validation *in vivo* from mice to non-human primates (toxicity, immune response, safety, and protection). Finally, the vaccine candidate is tested in human subjects in clinical trials. In phase I, safety is evaluated in a small group of healthy volunteers; in phase II, safety and immunogenicity are evaluated in a few hundreds of healthy volunteers; and in phase III, safety and efficacy are evaluated in thousands of healthy volunteers (Sette & Rappuoli, 2010). All product and trial data are revised by the regulatory agency for registration and once approved, early manufacturing may start. Post-approval, the vaccine is released to the public and phase IV (pharmacovigilance) continues to monitor safety and efficacy post-commercialization in real-world conditions (Figure 1) (Funk et al., 2020).

The cost of studies from preclinical to completion of clinical trials can reach billions of dollars (Gouglas et al., 2018) and the average time required from phase I to regulatory review varies from 6-11 years (Sette & Rappuoli, 2010). It is a challenging endeavor since most drugs and vaccine candidates will fail in some stage of clinical trials. Vaccines are comparatively the strategy most likely to succeed (Wong et al., 2019).

The unique scenario created by the pandemics has produced hundreds of vaccine candidates and led 8 of these up to phase III in only months. This remarkable achievement can be explained by the combination of a series of factors through the whole vaccine development process: 1) Scientifically, coronavirus were known pathogens, and important knowledge had been collected, especially from the SARS and MERS epidemics in 2003 and 2012, respectively. For instance, there was previous knowledge that the spike protein was a good antigen to be used in a vaccine. Additionally, the COVID-19 is an acute disease, meaning that the host's natural immune response may eliminate the pathogen. Analysis of this response provides important information of the type of immune response induced, important targets, etc; 2) Focused resources and joint efforts of collaborators and public-private partnerships shortened the crosstalk between academic studies and product development; 3) Combined phases of clinical trials and the proactive support of regulators enabled a faster authorization to move forward; 4) New generation vaccines e.g. RNA and viral vectors bring shorter production times, and accumulated experience with traditional technologies e.g. inactivated vaccines, will enable the accelerated establishment of production. Furthermore, producers are making their preparations “at-risk” before final evaluation in phase III. This involves the construction/renovation of production facilities without even knowing if the candidate will move forward; 5) Funding and coordination from public-private initiatives enabled the acceleration of research. WHO is ensuring that all candidates have the chance to be tested within 3-6 months, in the Solidarity Call for Action (WHO, 2020b). The NIH Accelerating COVID-19 Therapeutic Interventions and Vaccines (ACTIV) is a public-private partnership to coordinate the research (NIH, 2020). The Coalition for Epidemic Preparedness Innovations (CEPI) (Lurie et al., 2020), the Biomedical Advanced Research and Authority Development (BARDA, 2020), the Global Alliance for Vaccines and Immunization (GAVI) (Berckley, 2020), and the Bill & Melinda Gates Foundation (Gates, 2020), have all been contributing to raise funds and provide supplies; 6) Millions of vials, syringes, and other supplies, along with an appropriate cold-chain are being secured to, as early as possible, bring the vaccines to the world. This accelerated development has raised concerns on how the safety of the vaccines will be guaranteed. But never a vaccine development process has been followed by so many “eyes”. While regulatory agencies are expediting the processes, it must be ensured that all results are shared, analysed and discussed by the scientific community (Figure 1).

## Worldwide initiatives on vaccine development

Several institutions across the world, from universities to biotechs and multinational pharmaceuticals are developing vaccine candidates for SARS-CoV-2. Exploring new and old technologies, these platforms can be classified by the type of vaccine into eight main cathegories: live attenuated, inactivated, protein subunit, DNA, RNA, non-replicative viral vector, replicating vector, and virus-like particles (VLP) plus a few others. At the time of writing, there are 11 vaccine candidates in phase III clinical trials, three others in phase II, 13 initiatives are in phase I/II and 21 in phase I. Aside from live attenuated vaccines, which have no proposal in clinical trials, all other platforms are represented among these candidates (Table 1)(WHO, 2020a).

### Inactivated vaccines

Inactivated vaccines use the causative agent of the disease in its inactivated form (Gao et al., 2020). Vaccines based on the whole virus require a cellular platform and are typically produced in cell culture (or eggs). Once harvested, the virus is inactivated through physical (e.g. gamma irradiation) or chemical processes (e.g. formaldehyde). This is a well-established approach currently used for several viral vaccines such as the inactivated poliovirus vaccine (IPV), rabies, and hepatitis A vaccines (CDC, 2020). Inactivated vaccines produce a potent immune response and are generally less reactogenic compared to live attenuated vaccines. Additionally, they have fewer regulatory obstacles for licensing (Zepp, 2010). 

CoronaVac, developed and produced by the Chinese company Sinovac, is an example of a classic vaccine. Pre-clinical studies in monkeys showed that CoronaVac induced a weak cellular response, with no notable changes in the production of cytokines by T cells, which displayed no pathology to the tissues of the lung, heart, spleen, liver, kidney, and brain. More importantly, increased production of virus-specific antibodies was observed and monkeys immunized with 6 μg/dose of CoronaVac showed complete protection against the SARS-CoV-2 challenge (Gao et al., 2020)(Table 1).

CoronaVac is currently in phase III clinical trial in Brazil and Indonesia. The huge effort of Instituto Butantan to produce the CoronaVac vaccine in its facilities should be highlighted. This will enable a considerable scale-up in the number of doses produced.

This type of vaccine also has other candidates in clinical trials. BBIBP-CorV (Beijing Institute of Biological Products/Sinopharm), was shown to induce high levels of neutralizing antibodies in mice, rats, guinea pigs, rabbits, and non-human primates (cynomolgus monkeys and rhesus macaques). In rhesus macaques, two doses of BBIBP-CorV (2 µg/dose) induced high levels of protection against intratracheal challenge with SARS-CoV-2. No evidence of antibody-dependent enhancement was observed. Regarding the manufacturing process, BBIBP-CorV showed high productivity and good genetic stability (Wang et al., 2020). BBIBP-CorV is in phase III in Abu Dhabi. Other clinical trials using inactivated vaccine candidates are being conducted by the Wuhan Institute/Sinopharm and Bharat Biotech which are all in phase III. Another three inactivated vaccine candidates are in phase I/II (Table 1). There are currently 15 other inactivated vaccine candidates in pre-clinical trials (WHO, 2020a). Overall, these are encouraging results. Combined with previous experience with many other inactivated vaccines available, these candidates show good projections for the inactivated vaccines against SARS-CoV-2.

### Non-replicating adenovirus vectors

Adenovirus comprise a family of double-stranded DNA viruses. They can infect a wide variety of hosts and, in humans, cause respiratory symptoms as those present in a common cold. The exploitation of adenovirus became initially popular for gene therapy but soon its use as vaccine vectors became evident for several reasons. Its genome is well characterized and relatively easy to manipulate. Most adenoviruses cause mild illness in immunocompetent human adults and, by excluding crucial regions of the genome, these vectors have a replication defect, which increases their predictability and reduces unwanted side effects (Tatsis & Ertl, 2004). Human (e.g. Ad5 and Ad26), chimpanzee (ChAd), and simian (e.g. GRAd) adenovirus are currently being investigated for COVID-19 as vaccine vectors. Human adenovirus has the advantage of being well suited for the human host (e.g. efficient cell transduction) but its efficiency can be impaired by pre-existing immunity against the vector. On the other hand, chimpanzee or other adenovirus vectors can circumvent this issue but their efficient delivery to the host cells must be confirmed (Alonso-Padilla et al., 2016).

The ChAdOx1 nCoV-19, later renamed AZD1222, was developed at the University of Oxford in a partnership with the pharmaceutical company AstraZeneca. ChAdOx1 stands for chimpanzee adenovirus-vectored vaccine. It is a non-replicating viral vector expressing SARS-CoV-2. One-dose of AZD1222 induced a robust humoral and cellular immune response in mice and Rhesus macaques, as demonstrated by specific IgG induced against the spike protein and expression of cytokines by T cells. Rhesus macaques immunized with 2.5 x 10^10^ PFU (plaque-forming units) of ChAdOx1 nCoV-19 showed antigen-specific antibodies as early as 14 days post-vaccination and endpoint IgG titers of 400-6,400 on the day of the challenge. Virus-specific neutralizing antibodies were detectable in all immunized animals before the challenge. After the challenge with SARS-CoV-2, a significant decrease in the viral load in bronchioalveolar lavages was observed as compared to non-immunized animals (van Doremalen et al., 2020)(Table 1). This vaccine candidate was also evaluated for the expression of adenoviral backbone genes in human cell lines (Abdulaziz et al., 2020). As adenovirus vectors advance in clinical trials this is an important consideration since it could promote generation of anti-vector immunity. This vaccine is currently in phase III clinical trial in the UK, India and in Brazill.

Another adenovirus-based vaccine candidate, Ad5-nCov (CanSino Biological), induced both humoral and cellular immune responses after 3 doses in a preliminary human trial. Although some adverse reactions were reported (Zhu et al., 2020), they were considered not severe and thus justified its progression to a proper clinical trial evaluating safety and immunogenicity in humans. This candidate recently entered phase III trials, as well as other formulations based on non-replicating viral vectors: Ad26.COV2-S (Janssen Pharmaceutical) and Gam-COVID-Vac (Gamaleya Research Institute). GRAd-COV-2 vaccine, investigated by a collaboration of Institut Pasteur/Thera/LEUKOCARE/Univercells is currently in phase I. Although most non-replicative viral vectors in development are adenovirus-based, there is also one candidate in clinical phase I based on Modified Vaccinia Ankara (MVA) developed by the University of Munich (Table 1). Additionally, another 19 proposals are using non-replicating viral vector-based vaccines in pre-clinical trials (WHO, 2020a).

### RNA-based vaccines

RNA-based vaccines represent a new generation of vaccines. They are constituted by the insertion of a messenger RNA (mRNA) containing the gene of the antigen of interest in the 5’- 3’ untranslated regions (for non-replicating vaccines). Alternatively, a self-amplifying mRNA will also comprise parts of the viral genome, that enables the formation of replication-defective viral particles. This is achieved by the presence of structure-related genes and the absence of all replication-related genes. These vaccines require a liposome-like structure for stabilization and delivery to the cells. Once the genetic material is introduced, target cells will produce the viral proteins and induce specific immune responses (Pardi et al., 2018). Using RNA, the sequence does not have the risk of being integrated into the genome. Other advantages include: easy design, no risk of anti-vector immunity (since mRNA is a minimal genetic vector), it allows repeated administration, and rapid manufacturing. However, there are no RNA vaccines approved for use in humans yet, and they can cause local inflammatory reactions; they also require a low-temperature chain (-70^o^C) to maintain stability.

The candidate vaccine mRNA-1273 (Moderna/NIAID) is an RNA-based vaccine. mRNA-1273 is a lipid nanoparticle-encapsulated, nucleoside-modified messenger RNA (mRNA) that encodes the SARS-CoV-2 spike (S) glycoprotein, stabilized in its prefusion conformation. Healthy adults in phase I clinical trials received two doses of either 25, 100, or 250 μg, 28 days apart. The vaccine-induced robust antibody responses, binding to both full-length S protein and receptor-binding domains in all participants after the first dose in a time- and dose-dependent manner. Neutralizing antibody responses were also induced in a dose-dependent manner. Seroconversion of binding antibodies occurred within 2 weeks after the first dose, but the neutralizing activity was only achieved after the second dose, supporting a two-dose vaccination schedule. Of the doses evaluated, the 100 μg dose induced high antibody neutralization responses and Th1-shifted CD4^+^ T cell responses, along with a reactogenicity profile that is more favorable than the highest dose (Jackson et al., 2020). Adverse events such as fatigue, chills, headache, myalgia, and pain at the injection site were more frequent and more severe with higher doses and after the second dose. This reactogenicity profile had previously been reported in the trials of avian influenza mRNA vaccines (influenza A / H10N8 and influenza A / H7N9), also manufactured by Moderna, but using an earlier lipid nanoparticle capsule formulation (Jackson et al., 2020).

The mRNA-1273 vaccine is in phase III in the United States as well as BNT162 (BioNTech/Fosun Pharma/Pfizer). Other RNA-based vaccine candidates in clinical trials are ARCT-021 (Arcturus/Duke-NUS), Covac 1 (Imperial College London), CVnCoV (Curevac), and an unnamed vaccine of the People’s Liberation Army-Academy of Military Sciences/Walvax Biotech (Funk et al., 2020; Pagliusi et al., 2020; WHO, 2020a)(Table 1). There are currently another 22 proposals for mRNA-based vaccines in the pre-clinical stage (WHO, 2020a).

### DNA-based vaccines

DNA-based vaccines also belong to a new generation of vaccines. The DNA encoding the antigen gene is introduced via a plasmid directly into the cells of a specific tissue, often facilitated by nano-carriers. Once the nucleic acid is captured by the cells, it will be transported to the nucleus and initiate protein expression; an immune response is expected to be induced against the synthesized protein. These vaccines are considered one of the safest approaches as they do not involve any handling of the pathogen, can induce robust immune responses, allow multivalent formulations, are stable and large quantities can be produced in a short time. However, an efficient delivery system is required and there are concerns on is potential risk of integration into the host cell genome. Although this strategy has been investigated for many years, there are no licensed vaccines for humans yet. However, there are high expectations in clinical trials with DNA vaccines for Ebola (Lambe et al., 2017), influenza (Lee et al., 2018), and Zika virus (Abbink et al., 2018), besides its use in immunotherapy (Lopes et al., 2019).

The INO-4800 vaccine (Innovio - plasmid pGX9501) comprises a DNA vaccine encoding the full length of the Spike glycoprotein of SARS-CoV-2. This plasmid is delivered to the cells by the platform called CELLECTRA, which uses a brief electrical pulse to reversibly open small pores in the cells allowing the plasmid to enter (Precision Vaccinations, 2020). INO-4800 vaccine induced a robust humoral and cellular immune response in mice and guinea pigs. Protein-specific IgG antibodies against SARS-CoV-2 were detected in bronchioalveolar lavages (BAL) (Smith et al., 2020). Rhesus macaques, receiving two intradermal immunizations with INO-4800 (1 mg) induced T cell responses and neutralizing antibody responses against SARS-CoV-2 spike proteins. The peak of T cell responses was detected two weeks after the second immunization and neutralizing antibodies after 12 weeks. The intranasal and intratracheal challenge with SARS-CoV-2 performed 13 weeks post-final immunization with 1.1 x 10^4^ PFU of SARS-CoV-2 showed a rapid recall response against multiple regions of the S protein. This response was characterized by the expansion of neutralizing antibody levels, as well as the rapid expansion of T cell responses (Patel et al., 2020). 

Several other institutions have led DNA-based vaccine candidates to clinical trials. These are Genexine Consortium (GX-19), Cadila Healthcare (ZyCoV-D), Osaka University/AnGes/Takara Bio (AG0301 - COVID19), and Symvivo (bacTRL-Spike) (Funk et al., 2020; Pagliusi et al., 2020; WHO, 2020a). With the exception of bacTRL-Spike, these DNA-based candidates are in clinical trials phase I/II (Table 1) and there are another 14 proposals in the pre-clinical stage (WHO, 2020a).

### Protein subunit vaccines

Protein subunit vaccines rely on the use of an isolated antigen from the pathogen. Subunit vaccines are very safe. Since they contain only a few antigens or fragments, there is no need to handle the pathogen, and they can constitute multi-antigen platforms. However, they are generally weaker in immunogenicity than live-attenuated vaccines, requiring strong adjuvants, and production-associated issues e.g. in protein folding or incorrect glycosylation are common. For SARS-CoV-2, the most explored antigen is the structural protein, spike (S), or its receptor-binding domain (RBD), important for viral entry into the cells. Other proteins to be explored are the matrix, envelope, and nucleocapsid proteins. 

Novavax's NVX-CoV2373 is based on a recombinant protein expressed in insect cells and incorporated into a new nanoparticle (27.2 nm) formulated with saponin-based Matrix-M adjuvant. In mice and baboons, a low-dose of NVX-CoV2373 elicited high titers of anti-Spike IgG antibodies. It also induced CD4^+^ and CD8^+^ multifunctional T cells, CD4^+^ follicular T helper cells, and the generation of antigen-specific germline B cells in the spleen (Tian et al., 2020). In the clinical trial phase I, NVX-CoV2373 induced neutralization titers in 100% of participants after a second dose of the vaccine. This vaccine is currently in clinical trial phase III. Combination with Matrix-M™ adjuvant-induced robust polyfunctional CD4^+^ T cell responses (Novavax, 2020)(Table 1).

Other initiatives using subunit vaccines are in phase I/II clinical trials and are conducted by Anhui Zhifei Longcom Biopharmaceutical vaccine, which uses a dimer composed by two RBD domains (RBD-SC-dimer) (Dai et al., 2020), Kentucky Bioprocessing (KBP-201), Sanofi Pasteur / GSK (spike antigen expressed in baculovirus) and Biological E Ltd (BECOV). There are another 10 candidates in phase I and 55 in the pre-clinical stage (WHO, 2020a).

### Other approaches

There is one proposal based on a replicative viral vector in phase I clinical trial. It is based on the use of an attenuated measles virus as a vector to express the spike protein of SARS-CoV-2. This approach is being investigated by a collaboration between Institute Pasteur, Themis, Univ. of Pittsburg, and Merck. Themis Bioscience is investigating the use of attenuated measles virus as a vaccine vector for chikungunya. This vaccine is currently in phase II clinical trials and demonstrated safety, immunogenicity, and functionality of the technology in humans, even in the presence of pre-existing anti-measles immunity (Reisinger et al., 2019). Another interesting platform reaching clinical trials is the plant-derived VLPs by Medicago Inc, which uses VLPs combined with proprietary adjuvants from GSK or Dynavax (Table 1). These VLPs comprise the spike protein of SARS-CoV-2 presented in a lipid bilayer as true VLPs. Further description of VLPs is found in the next section. There are another 12 VLP-based vaccines in pre-clinical studies in 10 countries (WHO, 2020a).

Dendritic cells (DC) loaded with antigens from the SARS-CoV-2, artificial antigen-presenting cells (APC) altered by lentivirus, and genetically attenuated SARS-CoV-2 are other approaches currently in clinical trials (NCT04276896), but there is not much data available at the moment. A summary of information for all vaccine candidates in clinical trials is presented in Table 1.

## Brazilian initiatives on vaccine development

Brazilian efforts to develop a SARS-CoV-2 vaccine are also underway. Many established groups with extensive experience in vaccine development are refocusing their attention to COVID-19. Despite the difficulties with budget constraints and now the pandemics, each initiative investigates a different strategy and the first results are expected to be available soon (Table 2).

**Table 2 -:** Brazilian initiatives on vaccine development for COVID-19.

Leading Institution		Platform	Type	Properties	Collaboration	Similar platform in use
FIOCRUZ-MG	INCT- Vacinas^a^	Replicating virus vector	Influenza vaccine vector	Bivalent vaccine. Takes advantage of the established use of influenza vaccine	UFMG, USP (ICB, InCOR), Instituto Butantan, FMRP-USP	Seasonal influenza.
Instituto Butantan	LDV^b^	Conjugated	OMV coupled with antigen	Great adjuvanticity conferred by OMV	-	OMVs are component of Meningitis B (Bexsero)
	RCDV^c^	Subunit vaccines, VLPs and chimeras	-	-	Several laboratories in the Institution	-
UFSC	CCB (MIP)^d^	Live attenuated vector	Recombinant BCG	Established vaccine against Tuberculosis. Worldwide used. Bivalent vaccine.	UFMG, Instituto Butantan, UFRJ, Cambridge Univ., UKKarolinska Institutet (Sweden)	The vector is the current vaccine
USP	FMUSP (InCOR)^e^	VLP	VLP containing spike protein	Mimics the viral structure	UFMG, USP (ICB), UNIFESP	HPV
	ICB (LDV)^f^	Nanoparticles	Self-assembly protein nanoparticles (SAPN)	Mimics the viral structure. Flexibility in the choice of antigens	-	-
	FCF^g^	Nanoparticles (spray)	Chitosan nanoparticles coupled with antigen	Nasal administration	-	-
	Poli (PQI)^h^	Nanoparticles	Gold nanoparticles coupled with antigen	Custom size, shape and surface	USP (ICB)	-
UFV	DBG^i^	Replicating virus vector	Yellow fever vaccine vectored	Established vaccine. Bivalent vaccine	UFV (DEM, DMB), FIOCRUZ-PE	The vector is the current vaccine

^a^ Instituto Nacional de Ciência e Tecnologia - Vacinas; ^b^ Laboratório de Desenvolvimento de Vacinas; ^c^ Rede Colaborativa de Desenvolvimento de Vacinas, ^d^ Microbiologia, Imunologia e Parasitologia do Centro de Ciências Biológicas (Universidade Federal de Santa Catarina); ^e^ Faculdade de Medicina da Universidade de São Paulo - Instituto do Coração; ^f^ Laboratório de Desenvolvimento de Vacinas do Instituto de Ciências Biomédicas; ^g^ Faculdade de Ciências Farmacêuticas; ^h^ Departamento de Engenharia Química da Escola Politécnica; ^i^ Departamento de Biologia Geral (Universidade Federal de Viçosa).

### VLPs

In Brazil, studies with VLPs are being conducted by the Instituto do Coração (InCor) in a broad collaboration with other institutions. VLPs are composed of multiple viral proteins required to form the viral structure but do not contain the genetic material. This strategy ensures the safety of the approach since VLPs cannot replicate in the host. The similarities with a real virus allow VLPs to elicit both humoral and cellular immune responses (Syomin & Ilyin, 2019); VLPs are usually more immunogenic than the respective recombinant proteins. The researchers currently conducting this approach for SARS-CoV-2 have previously employed the VLP strategy as a vaccine for Zika virus (ZIKV). Using a modified Cucumber mosaic virus (CuMVtt) chemically coupled with E-DIII (domain III of ZIKV E protein), the vaccine increased the IgG antibody levels, mainly of the IgG2 isotype. More importantly, immunized mice induced neutralizing antibodies with no indication of antibody-dependent enhancement (ADE) of infection (Cabral-Miranda et al., 2019)(Table 2).

A key aspect of this strategy lies in the fact that very successful vaccines against other diseases are based on VLPs. Historically, the first human vaccine based on recombinant DNA technology was a VLP. Although the Hepatitis B antigen (HBsAg) can self-assemble forming these structures, they do not resemble an intact virion and therefore are not considered as “true” VLPs. These Hepatitis B vaccines, Recombivax-HB (Merck) and Engerix-B (GSK), were introduced in the late 1980s. Twenty years later, the vaccines against the human papillomavirus are VLP-based and their robustness is supported by the availability of not one but two HPV vaccines produced by different companies, Gardasil (Merck) and Cervarix (GSK). The major capsid antigen from HPV is either expressed in yeast or insect cells. While each system has its inherent challenges, it certainly shows the flexibility for the production of recombinant antigens. Today, many vaccine candidates based on VLPs for influenza, parvovirus, hepatitis, and malaria are undergoing clinical trials (Roldao et al., 2010).

### Influenza vector

The influenza virus can be genetically engineered to contain in its structure antigens from other pathogens. Studies conducted at Instituto René Rachou, Fiocruz in Minas Gerais, uses a platform based on an attenuated recombinant influenza vector to express antigens of medically important pathogens. Previous studies have demonstrated that the influenza A virus (IAV) harboring a truncated neuraminidase gene is safe and can induce protective immunity against influenza virus challenge in mouse models (Barbosa et al., 2014). The group used this strategy in a heterologous prime-boost regimen with the influenza vector as prime and an adenovirus vector boost, both carrying the ASP2 antigen of *Trypanosoma cruzi*. Mice immunized with this vaccine displayed augmented *T. cruzi* epitope-specific CD8+ T cells and showed increased survival rates when challenged with *T. cruzi* (Barbosa et al., 2013). A recombinant IAV harboring the EDIII domain of West Nile Virus (WNV) induced specific T cells and antibodies in immunized mice and protection against WNV challenge. Passive transfer of serum or CD4^+^ T cells from immunized mice into naïve recipients promotes the control of WNV replication in the brain and decreased body weight loss (Martina et al., 2011).

Since 2006, the group has been studying vaccines for dengue, leishmaniasis, Chagas disease, and malaria using this platform. For SARS-CoV-2, the goal is to express a fragment of the spike protein and also the seasonal H1 antigen from the influenza virus. Ideally, this bivalent vaccine would protect against seasonal influenza and SARS-CoV-2. This effort is a collaboration that involves the Instituto Nacional de Ciência e Tecnologia - Vacinas (INCT - Vacinas) and other institutions in the country (Table 2). A recent review on the use of influenza A as a vaccine vector covers many of its applications, shows its advantages and concludes that this is a promising strategy in vaccine development (Gerlach et al., 2019).

### Outer membrane vesicles (OMV)

Different strategies are being pursued at Instituto Butantan. Our group at the Laboratório de Desenvolvimento de Vacinas will use OMVs efficiently coupled with SARS-CoV-2 antigens on its surface using the novel Multiple Antigen Presenting System (MAPS). The MAPS strategy, developed at Boston Children’s Hospital, Harvard (Zhang et al., 2013), was successfully applied in the development of a Pneumococcal vaccine, coupling polysaccharides, and recombinant proteins, being currently in clinical trials (NCT03803202). Our group has adapted the technology to couple recombinant proteins to OMVs derived from *Neisseria lactamica.* This involves the use of biotinylated OMVs and antigens expressed in fusion with avidin derivatives. The strong affinity of biotin-avidin naturally attaches the antigen to OMV. This approach enabled a marked increase in the antibody levels in immunized mice in comparison to the antigen not coupled to OMV or the simple mixture of the components (Patent application).

The Institution will additionally address the vaccine development for SARS-CoV-2 through the recently established Rede Colaborativa para Desenvolvimento de Vacinas (RCDV). This joint effort brings together many specialized laboratories to investigate subunit vaccines, VLPs, and chimeric proteins. Chimeras are an interesting approach. While inactivated or attenuated vaccines are considered highly immunogenic, they also contain many components that are not important to generate a protective immune response. Chimeras can combine exclusively the fragments targeted by the immune system to mount a protective immune response in a single protein. One of the challenges will be the control of proper folding of the antigens, since this new chimeric protein may also form new molecular interactions and alter its native conformation.

Furthermore, our group has extensive experience in recombinant BCG (rBCG) expressing antigens from other pathogens such as *Bordetella pertussis* (Kanno et al., 2019; Nascimento et al., 2000), *S. pneumoniae* (Goulart et al., 2017) or *Schistosoma mansoni* (Varaldo et al., 2004) having shown induction of appropriate immune responses and protection against challenge with the respective pathogens. Our group will be collaborating with the project coordinated by Universidade Federal de Santa Catarina (UFSC) towards the development of rBCG strains expressing SARS-CoV-2 antigens (see below).

### Recombinant BCG

Using BCG as a vector to express and present antigens is an attractive idea since BCG is a well-established vaccine all over the world. With almost a century since the first use in humans, BCG has an extensive history of safety. Additionally, it can be applied in newborns and is a potent adjuvant of the immune response (Kanno et al., 2019). The Departamento de Microbiologia, Imunologia e Parasitologia of UFSC coordinates a project to investigate recombinant BCG expressing different SARS-CoV-2 antigens. This project will be performed in collaboration with Universidade Federal de Minas Gerais (UFMG), our group at Instituto Butantan, Universidade Federal do Rio de Janeiro (UFRJ), Cambridge University, and the Karolinska Institute. Different fragments of the SARS-CoV-2 antigens based on the spike and nucleocapsid proteins, alone or as a chimera, will be expressed through a variety of mycobacterial expression vectors for the induction of neutralizing antibodies and a broad cellular immune response. One of the main issues regarding rBCG is to obtain an appropriate expression vector. To address this matter our studies also involved optimization of promoters strength by random mutagenesis (Kanno et al., 2016) and construction of stable expression vectors with different promoters (Nascimento et al., 2020). It is important to obtain different levels of expression since higher expressions of the antigen may not result in increased immune response or protection (Nascimento et al., 2017).

On the other hand, the BCG vaccine has shown immune-stimulation and protection against non-related pathogens, the so-called non-specific protection (trained immunity or heterologous protection). BCG can stimulate innate immune cells, such as macrophages and NK cells, to show an enhanced response to other pathogens (O'Neill & Netea, 2020). There are currently several clinical trials evaluating the heterologous protection conferred by BCG against SARS-CoV-2 (Giamarellos-Bourboulis et al., 2020) (NCT04384549, NCT04461379, NCT04327206, and NCT04328441), including studies in Brazil, at UNICAMP, Fiocruz, and others (NCT04369794). Other microorganisms that seem to induce non-specific protection, such as the Polio vaccine are also being studied (Netea et al., 2020).

### Self-assembling protein nanoparticles (SAPN)

Different from VLPs, which rely on antigens to form the viral structure, through SAPN the antigens can be genetically engineered into the sequence of the nanoparticle (Sanchez-Garcia et al., 2018; Unzueta et al., 2012). This approach gives flexibility to the system allowing the use of virtually any antigenic fragment. Interestingly, SAPN was already investigated for SARS-CoV. A fragment of the spike protein was displayed in its trimeric conformation by the use of oligomerization domains in fusion with the peptide of interest (Pimentel et al., 2009). The Laboratório de Desenvolvimento de Vacinas (LDV) at the Instituto de Ciências Biomédicas will use nanoparticles for vaccine development. The group is experienced in viral vaccines and has applied these developments to Dengue, Zika, and other flavivirus vaccines (Alves et al., 2016; Zaneti et al., 2019). The main strategy investigated for SARS-CoV-2 will be based on SAPN to produce modified SARS-CoV-2 antigens to enable their self-assembly and consequently mimic the architecture of the viral surface.

### Yellow fever virus vector

The SARS-CoV-2-YFV17D chimera strategy is based on the use of the YFV17D as a vector to express other antigens similarly to the use of measles virus vector already in phase I clinical trials. Another initiative in the pre-clinical stage also based on YFV17D is being pursued by Katholieke Universiteit (KU) Leuven demonstrating the feasibility of this approach (Felipe et al., 2020). A project for a chimera vaccine based on the yellow fever virus vaccine (YFV17D) is under investigation by researchers at the Universidade Federal de Viçosa (UFV). The group has experience in vaccine development having published previous work on dengue DNA vaccines (Calegari et al., 2016; De Paula et al., 2008).

### Other approaches

The Brazilian biotech company Farmacore, is developing, together with PDS Biotechnology (USA), a candidate vaccine named Versamune®-CoV-2FC. It combines a recombinant fusion protein from SARS-CoV-2 and Versamune, a cationic lipid nanoparticle with immune activation properties (PDS Biotechnology, 2020). Another strategy based on a chitosan nanoparticle carrying viral proteins to be applied as a nasal spray aiming at inducing mucosal IgA antibodies is also being investigated by the Faculdade de Ciências Farmacêuticas (Table 2).

## Conclusion

Every day new studies pave the way for novel vaccine approaches for SARS-CoV-2. While many vaccine candidates are well-advanced in clinical trials, the proportion of vaccines that fail in this last step is extremely high. The contributions of international institutions and the presence of well-financed pharma/biotech companies accelerate the research towards the goal of an efficient COVID-19 vaccine. The reality of a pandemic may disregard the cost of a vaccine, but full access will require lower pricing that may reduce the continuous interest of private companies. While this manuscript was under review several vaccines were approved and are now being applied in many countries including Brazil. Three platforms successfully passed phase III (RNA, viral vectors and inactivated virus) and others are still under evaluation. The incredible speed which these vaccines went through all clinical trials is unprecedent and hopefully will be used as an example of what can be accomplished when developers, industry, regulatory and funding agencies work together. Many of our regional infectious diseases do not call the same attention of private companies and international funding. The networks and complementary efforts established during the pandemics consolidate the collaborative environment created in Brazil and can be used to promote new developments to deal with regionally restricted problems, such as neglected tropical diseases. In the end, it may not only be a race for a COVID-19 vaccine but an advancement in the marathon for all other infectious diseases.
